# *Gynura procumbens* ethanolic extract suppresses osteosarcoma cell proliferation and metastasis *in vitro*

**DOI:** 10.3892/ol.2013.1315

**Published:** 2013-04-22

**Authors:** HENG WANG, JI WEN ZHOU, DA HUA FU, YANG ZHOU, WEN ZHAO CHENG, ZHI-LI LIU

**Affiliations:** 1 Department of Orthopedics, First Affiliated Hospital of Nanchang University, Nanchang, Jiangxi 330006, P.R. China; 2 Zhangzhou Health Vocational College, Zhangzhou, Fujian 363000, P.R. China

**Keywords:** *Gynura procumbens*, osteosarcoma, NF-κB, metastasis, chemotherapy

## Abstract

*Gynura procumbens* is a traditional herb used for the treatment of inflammation, rheumatism and viral infections, although the antitumor effect and its potential mechanisms of action remain unclear. In the present study, the antitumor effect of *Gynura procumbens* ethanolic extract (GPE) on the osteosarcoma (OS) cell line, U2-OS, was investigated *in vitro*. Cell proliferation and apoptosis were measured by 3-(4,5-dimethylthiazol-2-yl)-2,5-diphenyltetrazolium bromide (MTT) and flow cytometry assays, respectively. Transwell invasion and wound healing assays were performed to investigate the invasion and migration of the U2-OS cells. The results showed that GPE was able to inhibit U2-OS cell proliferation and metastasis and induce cell apoptosis. Furthermore, the expression of the NF-κBp65 protein was detected by western blotting to evaluate the effects of GPE on the nuclear transfer of NF-κB. It was demonstrated that the expression of the NF-κBp65 protein was significantly decreased by GPE. This indicated that GPE was able to inhibit the nuclear transfer of NF-κB. The study shows that GPE is able to induce apoptosis and suppress proliferation and metastasis in U2-OS cells via the inhibition of the nuclear translocation of NF-κB.

## Introduction

Osteosarcoma (OS) is the most common primary mesenchymal malignant tumor of the bone tissue in humans, particularly children and adolescents (1). It was not until the early 1970’s that the introduction of doxorubicin and methotrexate with leucovorin rescue showed promise for improving survival (2). Following the advent of effective chemotherapy, the five-year survival rate of patients treated with intensive multidrug chemotherapy and aggressive local control has been reported at 55–80% (1–3), although this figure has not noticeably improved in several years. Investigations into the reason for this are multifaceted and the main aspect is considered to be drug-resistance during chemotherapy (2).

*Gynura procumbens* is a decumbent perennial herb belonging to the Asteraceae family and is widely distributed in Southeast Asian countries, including China, Indonesia, Thailand and Malaysia. The stem and leaves of *Gynura procumbens* have been used as a food and traditional medicine, particularly in treating cancer, inflammation, rheumatism and viral infections (4,5). Pharmacological studies have shown that *Gynura procumbens* has anti-inflammatory (6), anti-hypertensive (7,8), anti-hyperglycemic (9,10), anti-herpes simplex virus (5), anti-oxidative (11) and anti-hyperlipidemic (12) effects. However, it is unclear whether *Gynura procumbens* has antitumor activities against OS, and its potential molecular mechanism is unknown.

Kim *et al* (13) revealed that the ethanolic extract of *Gynura procumbens* inhibited matrix metalloproteinase (MMP)-1 and MMP-9 expression induced by UV-B irradiation via the inhibition of pro-inflammatory cytokine mediator release and reactive oxygen species (ROS) production. It has been demonstrated that MMPs are essential in the degradation of the basement membrane and epimatrix, among which MMP-2 and MMP-9 are the most markedly correlated with tumor invasion and metastasis (14–19). MMP-2 and MMP-9 are overexpressed in osteosarcoma and promote osteosarcoma cell migration and invasion by degrading certain components of the basement membrane and epimatrix (20). A large number of studies have indicated that the activation and transposition of the NF-κB gene, an upstream gene of MMPs, are closely associated with tumor invasion and migration (19,21–23).

In the present study, the effect of *Gynura procumbens* ethanolic extract (GPE) on cell proliferation, apoptosis and metastais was analyzed in the OS cell line, U2-OS. Subsequently, the effect of GPE on the inhibition of the nuclear transfer of NF-κB in U2-OS cells was investigated.

## Materials and methods

### Plant material

Whole *Gynura procumbens* (Lour.) Merr. plants, excluding the roots, were collected from the Yifeng county of Jiangxi, China and authenticated at the Jiangxi University of Traditional Chinese medicine, China, by Professor Luo Guangming.

### Preparation of GPE

The leaves and stems from the fresh plant were cleaned and dried in an oven at 40°C, then ground into powdered form at 100 mesh size. A crude ethanolic extract was created by macerating the powder with 95% ethanol at 85°C for 12 h. The extract was concentrated until dry *in vacuo*, with a yield of 1.4%. The extract was then reconstituted in 95% ethanol and vacuum filtered. The resulting filtrate was subjected to evaporation *in vacuo*, which removed the ethanol and left an aqueous solution containing an ethanol-soluble precipitate. The GPE was dissolved in dimethyl sulfoxide (DMSO; 100 mg/ml) and the final concentration of DMSO in the culture medium was controlled at 0.1% (v/v).

### Cell lines and cell culture

The human OS cell line, U2-OS, was obtained from the American Type Culture Collection (Manassas, VA, USA) and routinely cultured in Dulbecco’s modified Eagle’s medium (DMEM; Hyclone, Waltham, MA, USA) supplemented with 10% fetal bovine serum (FBS; Sigma, St. Louis, MO, USA) in a humidified 37°C incubator containing 5% CO_2_.

### Cell growth assay

The U2-OS cell line was cultured in 96-well tissue culture plates at a cell density of 5,000 cells per well, in DMEM containing 10% FBS and 2 mM L-glutamine. Following adherence overnight, the medium was replaced and the cells were incubated with increasing concentrations (0, 5, 10, 20, 40, 80 and 160 *μ*g/ml) of GPE. Subsequent to treatment for 24 h, 3-(4,5-dimethylthiazol-2-yl)-2,5-diphenyltetrazolium bromide (MTT) assays were performed in triplicate at a wave-length of 490 nm.

### Flow cytometry (FCM)

Human OS U2-OS cells were seeded at 5×10^5^ cells/ml into T25 culture flasks for 24 h. The cells were then treated with 0, 10, 20, 40 and 80 *μ*g/ml GPE. Following incubation, the cells were trypsinized, washed with phosphate-buffered saline (PBS) and fixed overnight in ice-cold 70% ethanol. Subsequent to fixation, the cells were washed twice with 1% bovine serum albumin (BSA) in PBS, then resuspended in 1 ml DNA-binding propidium iodide (PI) solution (10 mg/ml in PBS, containing 0.05 mg/ml RNase A), incubated at room temperature in the dark for 15 min and analyzed with an EPICS XL flow cytometer (Beckman Coulter, Miami, FL, USA). The number of apoptotic cells were measured using the control software of the flow cytometer.

### Western blot analysis

U2-OS cells in the exponential growth phase were treated with various concentrations of GPE (0, 20 and 40 *μ*g/ml) for 24 h. The cells were then washed with cold PBS. Total protein from the cells was extracted using radio-immunoprecipitation assay (RIPA) lysis buffer containing 60 *μ*g/ml phenylmethanesulfonylfluoride (PMSF) and the protein concentration was determined using a Bradford assay. Equal amounts of protein were electrophoresed by 10% SDS-PAGE and transferred onto a pure nitrocellulose blotting membrane (0.22-*μ*m pores). The membranes were blocked with 5% Difco skimmed milk for 1 h at room temperature (RT), then blocked with primary antibody (rabbit anti-NF-κBp65 IgG; 1:2,000; Santa Cruz Biotechnology, Inc., Santa Cruz, CA, USA) overnight at 4°C. The membranes were then washed prior to incubation with the appropriate peroxidase-conjugated secondary antibodies (anti-rabbit, 1:5,000; Santa Cruz Biotechnology, Inc.). The immune complexes were detected with a Pro-light HRP kit (Tiangen, Beijing, China). All experiments were repeated six times.

### Invasion assay

The invasiveness of the U2-OS cells was measured using BD BioCoat™ BD Matrigel TM Invasion Chambers (BD Bioscience, Franklin Lakes, NJ, USA) according to the manufacturer’s instructions. The medium in the lower chamber contained 5% fetal calf serum as a source of chemoattractant. The cells were suspended in serum-free medium containing various concentrations of GPE (0 or 40 *μ*g/ml) and added to the upper chambers simultaneously (2×10^4^ cells/ml in 0.1 ml). The cells that passed through the Matrigel-coated membrane were stained with Diff-Quik (Sysmex, Kobe, Japan) and images were captured. Cell migration was quantified by direct microscopic visualization and counting. The values for invasion were obtained by counting three fields per membrane and the results are presented as the average of six independent experiments performed over multiple days.

### Migration assay

Cell migration was assessed by determining the ability of the cells to move into a cellular space in a two-dimensional *in vitro* wound healing assay. In brief, the cells were grown to confluence in six-well tissue culture plastic dishes to a density of ∼5×10^6^ cells/well. Subsequent to being treated with various concentrations of GPE (0 or 40.0 *μ*g/ml) for 24 h, the cells were denuded by dragging a rubber policeman (Fisher Scientific, Hampton, NH, USA) through the center of the plate. The cultures were rinsed with PBS and fresh quiescent medium alone or with 10% FBS was added, after which the cells were incubated at 37°C for 24 h. The cells were photographed at 0 and 24 h and the migrated distance was measured. The cell migration rate was obtained by counting three fields per area and the results presented as the average of six independent experiments performed over multiple days.

### Statistical analysis

Data are expressed as the mean ± SD. The differences in invasion and migration between cells treated with GPE and the control group were evaluated using independent-sample t-tests. P<0.05 was considered to indicate a statistically significant difference. All analyses were performed using SPSS Version 13.0 (SPSS Inc., Chicago, IL, USA).

## Results

### Effect of GPE on U2-OS cell proliferation in vitro

**Figure 1.**
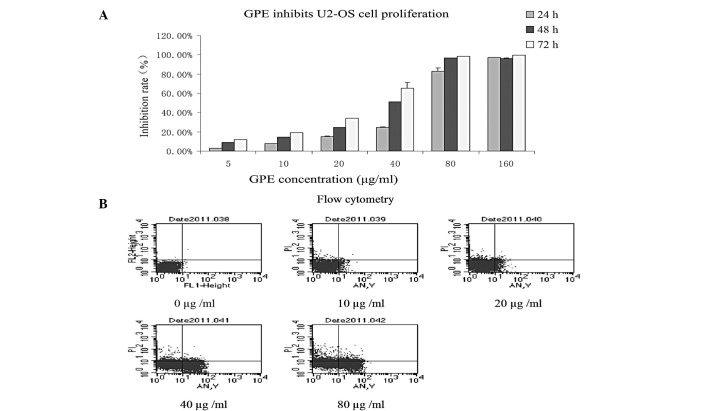
(A) GPE suppresses U2-OS cell proliferation. Six MTT assays were performed using U2-OS cells at 24, 48 and 72 h. The inhibition rate was increased with the GPE concentration and treatment time, indicating that GPE inhibits U2-OS cells proliferation in a dose- and time-dependent manner. (B) GPE induces U2-OS cell apoptosis. There were few apoptotic cells in the control group (0 *μ*g GPE). The percentage of apoptotic cells was 5.5% when treated with 10 *μ*g/ml GPE and 7.6% when treated with 20 *μ*g/ml GPE. The percentage of apoptotic cells increased to 24.7% following treatment with 40 *μ*g/ml GPE. The percentage of apoptotic cells increased further to 37.94% following treatment with 80 *μ*g/ml GPE. The results suggested that GPE induced U2-OS cell apoptosis. GPE, *Gynura procumbens* ethanolic extract; OS, osteosarcoma; MTT, 3-(4,5-dimethylthiazol-2-yl)-2,5-diphenyltetrazolium bromide.

The effect of GPE on the growth of the U2-OS cell line was investigated using MTT assays. The growth curves indicated that the U2-OS cells were sensitive to GPE and that growth inhibition occurred in a dose- and time-dependent manner (Fig. 1A), indicating that GPE was able to inhibit U2-OS cell proliferation *in vitro*.

### GPE induces U2-OS cell apoptosis

FCM analysis was used to investigate the effect of GPE in inducing U2-OS cell apoptosis *in vitro*. GPE at various concentrations was added to the U2-OS cell cultures in the exponential growth phase. Subsequent to treatment for 24 h, treated and untreated cell samples were obtained and fixed for FCM analysis. The FCM analysis demonstrated that the percentages of apoptotic cells were 0, 5.5, 7.6, 24.7 and 37.94% at 0, 10, 20, 40 and 80 *μ*g/ml GPE. This indicates that apoptosis occurred in a dose-dependent manner in cells treated with GPE (Fig. 1B) and GPE was able to induce U2-OS cell apoptosis *in vitro*.

### GPE inhibits U2-OS cell invasion in vitro

**Figure 2.**
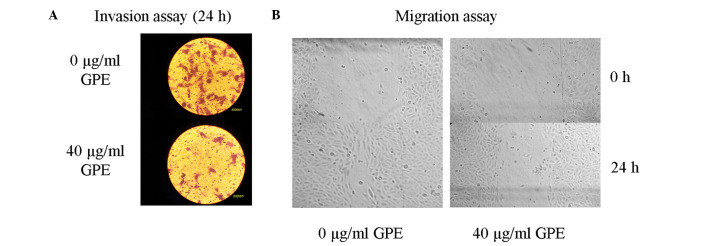
(A) GPE suppresses U2-OS cell invasion. A representative image from six experiments is shown (crystal violet; magnification, ×400). The cell invasion of the U2-OS cells was inhibited by GPE (40 *μ*g/ml), indicating that GPE inhibits U2-OS cell invasion *in vitro*. (B) GPE inhibits U2-OS cell migration. A representative image from six experiments is shown for each group (magnification, ×400). The migration rate of the cells treated with 40 *μ*g/ml GPE was significantly lower compared with the control group, indicating that GPE suppresses U2-OS cell migration *in vitro*. GPE, *Gynura procumbens* ethanolic extract; OS, osteosarcoma.

The appropriate GPE concentration for the transwell invasion cell assay was determined according to the IC_50_ value. Invasion was measured using a transwell assay to analyze the effect of GPE on the invasiveness of the U2-OS cells (Fig. 2A). The cells were treated with 40 *μ*g/ml GPE for 24 h. The results showed that the invasion of the cells treated with GPE was significantly inhibited compared with the untreated cells, suggesting that GPE was able to suppress U2-OS cell invasion.

### GPE inhibits U2-OS cell migration in vitro

The appropriate GPE concentration for the migration assay was determined according to the IC_50_ value. Migration was measured using a migration assay to investigate the effect of GPE on the invasion of the U2-OS cells. The cells were treated with 40 *μ*g/ml GPE for 24 h. The results showed that the cell migration of the cells treated with GPE was significantly inhibited compared with the untreated cells, indicating that GPE was able to suppress U2-OS cell migration (Fig. 2B).

### GPE suppresses the nuclear transfer of NF-κB

**Figure 3.**
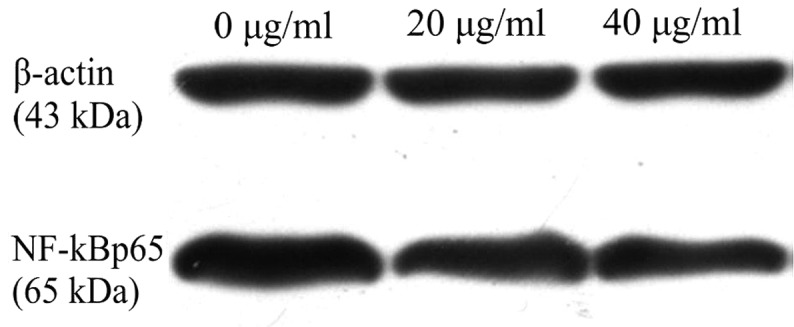
GPE suppresses the nuclear transfer of NF-κB. A representative image of six experiments is shown for each group. The expression of NF-κBp65 protein was suppressed following GPE exposure (20 or 40 *μ*g/ml for 24 h), indicating that the nuclear transfer of NF-κB was inhibited by GPE in the U2-OS cells. GPE, *Gynura procumbens* ethanolic extract; OS, osteosarcoma.

To investigate the effect of GPE on the nuclear transfer of NF-κB, the protein expression level of NF-κBp65 was detected. The results showed that NF-κBp65 protein expression was decreased significantly in the cells treated with GPE for 24 h compared with the untreated cells (Fig. 3), suggesting that GPE was able to inhibit the nuclear transfer of NF-κB in the U2-OS cells.

## Discussion

OS is the most common primary bone tumor in children and adolescents, with a five-year disease free survival rate of 70%. However, this figure has not noticeably improved over the past several years, mainly due to drug-resistance during chemotherapy and metastasis of the disease. Hence, it is necessary to develop novel therapeutic agents.

Herbal medicine is gaining popularity in developing countries. *Gynura procumbens* (Lour.) Merr., which is also known as ‘Sambung nyawa’, is widely used in Southeast Asian countries as a herbal medicine in the traditional treatment of numerous ailments, including eruptive fevers, rashes, kidney disease, migraines, constipation, hypertension and diabetes mellitus. The benefits of the traditional use of *Gynura procumbens* have also been supported by the isolation and identification of several possible active chemical components from this plant, including flavonoids, saponins, tannins and terpenoids (10). However, there is little evidence with regard to the anticancer activity of *Gynura procumbens*. In the present study, U2-OS cells were treated with GPE at various concentrations and the proliferation and apoptosis were measured by MTT and FCM analysis to investigate the effects of *Gynura procumbens* on tumor cell proliferation and apoptosis, respectively. The results demonstrated that cell proliferation was inhibited by GPE in a dose- and time-dependent manner and the rate of apoptosis was increased in cells treated with GPE, indicating that GPE was able to induce apoptosis and inhibit proliferation in U2-OS cells. Additionally, transwell invasion and migration assays were performed to evaluate the effect of GPE on U2-OS cell invasion and migration. The results revealed that the invasion and migration abilities of cells treated with GPE were significantly lower compared with cells that were not treated with GPE. This suggested that GPE was able to inhibit U2-OS cell invasion and migration *in vitro*.

The potential molecular mechanism of the inhibition of U2-OS cell invasion and migration by GPE remains unclear. Kim *et al* (13) reported that the ethanolic extract of *Gynura procumbens* inhibited MMP-1 and MMP-9 expression induced by UV-B irradiation via the inhibition of proinflammatory cytokine mediator release and ROS production. MMPs are enzymes that are directly responsible for the degradation of extracellular matrix (ECM) components, such as collagen and elastin. The degradation of ECM components has a significant role in tumor cell metastasis (24). Among all the MMPs, MMP-2 and MMP-9 are recognized as being particularly involved in the degradation of ECM components (14–19). The primary form of NF-κB is a heterodimer of the p50 and p65 subunits, which is localized mainly in the cytoplasm in an inactive form bound to an inhibitory protein named IκB (25). It has been shown previously that NF-κB upregulates MMP-9 (22), while the inhibition of NF-κB downregulates MMP-2 (23). In the present study, attempts were made to investigate whether GPE was able to regulate the nuclear translocation of NF-κB, resulting in the deregulation of the expression of MMPs in OS cells. The effect of GPE on the nuclear translocation of NF-κB was measured by detecting the expression of the NF-κBp65 protein. From the western blotting analysis, it was observed that the NF-κBp65 protein expression level was significantly lower in the U2-OS cells treated with GPE compared with the untreated cells. These results indicate that *Gynura procumbens* extract may inhibit the nuclear translocation of NF-κB.

In conclusion, the present study showed that GPE was able to significantly inhibit U2-OS cell proliferation and metastasis *in vitro* and that the inhibition of the nuclear translocation of NF-κB appeared to be the potential molecular mechanism. However, previous studies have shown that the tumor micro-enviroment may affect tumor cell proliferation, invasion and migration. Consequently, further *in vivo* experiments are necessary to confirm the anticancer activity of *Gynura procumbens* extract. Based on the results observed in the present study, it appears that further advances in the identification of the active principles of GPE are likely to provide more solid evidence of *Gynura procumbens* as an antitumor agent.
